# Epigenetic alteration at the *DLK1-GTL2* imprinted domain in human neoplasia: analysis of neuroblastoma, phaeochromocytoma and Wilms' tumour

**DOI:** 10.1038/sj.bjc.6602478

**Published:** 2005-03-29

**Authors:** D Astuti, F Latif, K Wagner, D Gentle, W N Cooper, D Catchpoole, R Grundy, A C Ferguson-Smith, E R Maher

**Affiliations:** 1 Department of Paediatrics and Child Health, Section of Medical and Molecular Genetics, University of Birmingham, The Medical School, Edgbaston, Birmingham B15 2TT, UK; 2 Cancer Research UK Renal Molecular Oncology Research Group, University of Birmingham, The Medical School, Edgbaston, Birmingham B15 2TT, UK; 3 TBA; 4 Department of Paediatric Oncology Birmingham Children's Hospital and Department of Paediatrics and Child Health, University of Birmingham, B15 2TT; 5 Department of Anatomy, University of Cambridge, Downing Street, Cambridge CB2 3DY, UK

**Keywords:** imprinting, DLK1, GTL2, methylation

## Abstract

Epigenetic alterations in the 11p15.5 imprinted gene cluster are frequent in human cancers and are associated with disordered imprinting of insulin-like growth factor (*IGF*)*2* and *H19*. Recently, an imprinted gene cluster at 14q32 has been defined and includes two closely linked but reciprocally imprinted genes, *DLK1* and *GTL2*, that have similarities to *IGF2* and *H19*, respectively. Both *GTL2* and *H19* are maternally expressed RNAs with no protein product and display paternal allele promoter region methylation, and *DLK1* and *IGF2* are both paternally expressed. To determine whether methylation alterations within the 14q32 imprinted domain occur in human tumorigenesis, we investigated the status of the *GTL2* promoter differentially methylated region (DMR) in 20 neuroblastoma tumours, 20 phaeochromocytomas and, 40 Wilms' tumours. Hypermethylation of the *GTL2* promoter DMR was detected in 25% of neuroblastomas, 10% of phaeochromocytoma and 2.5% of Wilms' tumours. Tumours with *GTL2* promoter DMR hypermethylation also demonstrated hypermethylation at an upstream intergenic DMR thought to represent a germline imprinting control element. Analysis of neuroblastoma cell lines revealed that *GTL2* DMR hypermethylation was associated with transcriptional repression of *GTL2*. These epigenetic findings are similar to those reported in Wilms' tumours in which *H19* repression and DMR hypermethylation is associated with loss of imprinting (LOI, biallelic expression) of *IGF2*. However, a neuroblastoma cell line with hypermethylation of the *GTL2* promoter and intergenic DMR did not show LOI of *DLK1* and although treatment with a demethylating agent restored *GTL2* expression and reduced *DLK1* expression. As described for *IGF2*/*H19*, epigenetic changes at *DLK1*/*GTL2* occur in human cancers. However, these changes are not associated with *DLK1* LOI highlighting differences in the imprinting control mechanisms operating in the *IGF2-H19* and *DLK1-GTL2* domains. GTL2 promoter and intergenic DMR hypermethylation is associated with the loss of *GTL2* expression and this may contribute to tumorigenesis in a subset of human cancers.

Genomic imprinting is a process whereby imprinted genes demonstrate parent-of-origin differences in allelic expression. Although only a minority of genes are imprinted, many of those identified to date have a role in the regulation of cell growth and differentiation and aberrant imprinting frequently leads to abnormal pre- and/or postnatal growth (Reik and Walter, 2001). Furthermore, disordered imprinting has been implicated in the pathogenesis of paediatric and adult cancers. Imprinted genes frequently occur in clusters. In particular, the imprinted gene cluster at 11p15.5, which contains the paternally expressed insulin-like growth factor (*IGF*)*2* and the maternally expressed *CDKN1C* and *H19* genes has been implicated in disorders of growth and in neoplasia (Maher and Reik, 2000; Feinberg *et al*, 2002). For example, germline-inactivating mutations in *CDKN1C* and epigenetic alterations leading to the loss of imprinting (LOI) (biallelic expression) of *IGF2* and silencing of *CDKN1C* or *H19* cause Beckwith–Wiedemann syndrome, a congenital overgrowth disorder associated with susceptibility to embryonal tumours (Maher and Reik, 2000; Weksberg *et al*, 2003). In addition, epigenetic alterations at *IGF2* and *H19* have been implicated in the pathogenesis of sporadic childhood (e.g. Wilms' tumour) and adult (e.g. colorectal) cancers (Cui *et al*, 2001; Nakagawa *et al*, 2001). *IGF2* and *H19* are closely linked and reciprocally imprinted, and epigenetic alterations in human cancers at an intergenic differentially methylated region (DMR) are associated with LOI of *IGF2* and silencing at *H19*.

In the past decade, the imprinted gene clusters at 11p15.5 and 15q13 (Prader-Willi and Angelman syndrome region) have been the subject of intense investigation. Recently, an imprinted gene cluster at 14q32 (distal mouse chromosome 12) was identified. The first imprinted 14q32 gene identified was *GTL2*/*Gtl2*, which, like *H19*, is a maternally expressed RNA with no apparent open reading frame (Miyoshi *et al*, 2000). Subsequently, a paternally expressed gene, *DLK1*/*Dlk1*, which encodes an EGF-like membrane protein similar to the *Drosophila Delta* gene was identified upstream of *GTL*/*Gtl2* (Kobayashi *et al*, 2000; Schmidt *et al*, 2000; Takada *et al*, 2000; Wylie *et al*, 2000). There are a number of similarities between the *IGF2*/*H19* and the *DLK1*/*GTL2* domains. In both cases, two closely linked genes are similarly organised, reciprocally imprinted and developmentally regulated; *H19* and *GTL2* do not encode known proteins, and paternally methylated DMRs associated with both genes have been implicated in the regulation of their imprinting (Paulsen *et al*, 2001; Takada *et al*, 2002).

In sheep, a single-nucleotide (nt) substitution is associated with altered postnatal expression of *GTL2* and *DLK1*, although their imprinting is not perturbed. This mutation results in the Callipyge muscle hypertrophy phenotype (Charlier *et al*, 2001; Freking *et al*, 2002; Georges *et al*, 2003). In human mutations or epigenetic alterations of *DLK1* and *GTL2* have not been described, although distinct phenotypes associated with maternal and paternal disomy for chromosome 14 are reported (Georgiades *et al*, 2000; Eggermann *et al*, 2001; Kurosawa *et al*, 2002). SAGE analysis demonstrated high expression of *DLK1* in a subset of neuroblastoma (van Limpt *et al*, 2000), but a follow-up study did not find any genetic alterations (e.g. mutation, duplication or rearrangement) or LOI in neuroblastomas with *DLK1* overexpression (van Limpt *et al*, 2003). In addition, downregulation of *GTL2* has been reported in pituitary adenomas with ectopic expression of *GTL2* inhibiting tumour growth *in vitro*, suggesting a tumour suppressor function for that gene (Zhang *et al*, 2003).

We have investigated neuroblastoma and other tumours to determine whether epigenetic alterations within the *DLK1-GTL2* imprinted domain are a feature of human neoplasia. We investigated neuroblastoma and phaeochromocytoma as *DLK1* is highly expressed in the adrenal medulla. Neuroblastoma may also demonstrate 14q allele loss and Wilms' tumour was of interest as epigenetic alterations at the *IGF2/H19* locus are common in this tumour.

## MATERIALS AND METHODS

### Multiplex methylation polymerase chain reaction (mPCR) assay

Methylation-specific PCR with oligonucleotide primers specific for the methylated and unmethylated copies of the *GTL2* promoter on chromosome 14q32 were performed using previously published primers (Murphy *et al*, 2003): MSM-UF (5′-GAG GAT GGT TAG TTA TTG GGG T-3′; nt position 65912 (GenBank accession no. AL117190)); MSM-UR (5′-CCA CCA TAA CCA ACA CCC TAT AAT CAC A-3′; nt 65931–66004); MSM-MF (5′-GTT AGT AAT CGG GTT TGT CGG C-3′; nt 64450–64471) and MSM-MR (5′-AAT CAT AAC TCC GAA CAC CCG CG-3′; nt 64587–64609). The multiplex mPCR conditions used were initial denaturation at 95°C for 3 min; five cycles of 95°C for 30 s, 70°C for 30 s, 72°C for 30 s; five cycles of 95°C for 30 s, 65°C for 30 s, 72°C for 30 s; followed by 30 cycles of 95°C for 30 s, 60°C for 30 s, 72°C for 30 s and a final extension of 72°C for 5 min. PCR amplification were performed using 0.05 U *μ*l^−1^ *Taq* polymerase in (NH_4_)_2_SO_4_ buffer with 3.0 mM MgCl_2_ (MBI Fermentas, St Leon-Rot, Germany). PCR products (160 bp for methylated allele and 120 bp for unmethylated) were separated on a 3% agarose gel, stained with ethidium bromide and visualised under UV illumination.

### Analysis of DNA methylation using bisulphite sequencing

Genomic DNA was treated with sodium bisulphite using previously published method (Herman *et al*, 1996). DNA (0.5–1 *μ*g) was denatured at 37°C for 10 min in 0.3 M NaOH followed by sulphonation of unmethylated cytosines by incubation in 3.12 M sodium bisulphite containing 1 M hydroquinone (pH 5) at 95°C for 30 s and 50°C for 15 min for 20 cycles. The resulting sulphonated DNA was purified using the Wizard DNA clean-up system (Promega, Southampton, UK) according to the manufacturer's instructions and DNA was eluted with 50 *μ*l of distilled water. Following elution, DNA was desulphonated in 0.3 M NaOH for 5 min at room temperature, ethanol precipitated and resuspended in 50 *μ*l distilled water.

### Methylation status of *GTL2* and *DLK1* DMRs

Following multiplex PCR methylation screening (see above), the methylation status of three areas of the CpG-rich region upstream of *GTL2* were analysed in detail (G1, position 65897–66197; G2, position 66541–66920; G3, position 67541–67910; GeneBank accession no. AL117190; relative to *GTL2* transcription start site, G1=−363 bp → −161 bp upstream; G2=+293 bp → +673 bp downstream; G3=+1213 bp → +1583 bp downstream *GTL2* transcription start site; GeneBank accession no. AY314975) and a region upstream *DLK1* promoter (D1, position 140551–140810; GeneBank accession no. AL132711) and two downstream regions (D2, position 141271–141420; D3, position 141571–141894; GeneBank accession no. AL132711). Except for D1 and D2 regions, bisulphite-modified DNA was amplified using two rounds of nested PCR (95°C for 15 min followed by 35 cycles of 95°C for 30 s, 55–58°C for 30 s, 72°C for 30 s and a final extension of 72°C for 5 min) using HotStar *Taq* DNA polymerase (Qiagen, Crawley, UK). Primer sets were designed to amplify DNA fragments containing both methylated and unmethylated CpG dinucleotides. The primer sequences are: G1F (5′-TTA GGT GTG GGA TTT GYG TTT YGA TAG TT-3′); G1R (5′-CAA AAA AAA TAA TCT CTA ACR TCA ACR CAT TCT ACT A-3′); G1FN (5′-GGT TAT TGG TYG TTT GAG GAY GGT TAG TT-3′); G2F (5′-TTA GGG TTT TTT TTT GGA GGG TTT AGT-3′); G2R (5′-AAA ACT AAT CCA TAA AAA CTA CTA ACA AAT-3′); G2RN (5′-ACC TAA AAT CCA CAC TAC ACT AAA CCT ATA-3′); G3F (5′-AGA GGG AAT AGT TTT GAG ATT TTT YGG ATT TAT-3′); G3R (5′-ATC CTC CAA ACA CCR CTA TCA CRC ATA TAA-3′); G3RN (5′-ATA ATC TCR AAA CRA AAA ACA AAA CCT ATA-3′); D1F (5′-TAT ATA GTG GGT ATT TTA ATT GTT TTT TAT-3′); D1R (5′-TAA AAA AAC AAA CCC ATA AAC ATC CCC AAA-3′); D2F (5′-TTG GTA ATT AGT ATT TTT TAT TTT TA-3′); D2R (5′-ACT TTT ATC ACA AAT AAC ATA CAT AAA C-3′); D3F (5′-TTG TTT ATG TAT GTT ATT GTG GAT-3′); D3R (5′-TAA AAT CCC RAA CAC ACR TAC AAT AAT-3′); D3FN (5′-GTT AAG GTT TTG ATT GAG ATG TTG TGT G-3′); and D3RN (5′-TAT ACC CCT AAC CAT AAA AAC RCA AA-3′). Amplification products were purified from agarose gel using QiaQuick gel extraction kit (Qiagen, Crawley, UK), cloned into pGem T-Easy vector system (Promega, Southampton, UK). In all, eight to 10 individual clones for each DMR regions were sequenced using BigDye terminator cycle sequencing kit V 1.1/3.1 (Perkin-Elmer/Applied Biosystems, Warrington, UK) and run on an ABI377/3730.

### Methylation status of the intergenic germline-derived differentially methylated region (IG-DMR)

A seminested PCR was performed to amplify the IG-DMR (nt position 51021–51180; GeneBank accession no. AL117190). The primer sequences are: IG-F (5′-TTT TGA GGA GAT TGA TAT TTT TAG TTT TAT T-3′); IG-R (5′-ATA AAC TAC ACT ACT AAA AAC TAC ATT TAA A-3′); and IG-Fnes (5′-TTA GGT TGG AAT TGT TAA GAG TTT GTG GAT T-3′). PCR was performed as previously described using HotStar *Taq* DNA polymerase (Qiagen) with an annealing temperature of 53°C and 1.5 mM MgCl_2_ for both first-round (primer set IG-F/IG-R) and second-round (primer set IG-Fnes/IG-R) PCRs. PCR products were then cloned and sequenced as described above.

### Expression analysis: reverse transcriptase (RT) and quantitative real-time PCR

RNA (1 *μ*g) was reverse transcribed using Reverse Transcription Systems and oligo-dT primers (Promega) according to the manufacturer's protocols. cDNA (1 *μ*l) obtained was then used as template for RT–PCR amplification. A single-nucleotide polymorphism (SNP) previously identified in exon 5 of *GTL2* and an SNP in exon 5 of *DLK1* (Wylie *et al*, 2000) were used to analyse *GTL2* and *DLK1* gene expression. *GTL2* and *DLK1* cDNA were amplified using oligonucleotide primers described by Wylie *et al* (2000), except that for *GTL2* reverse primer, GTL2RK (5′-TTC CAC GGA GTA GAG CGA GTC A-3′), was used. Amplification products were purified and sequenced as described above using primers: GTL2FS (5′-ATC CCT TTG GGA AAT TCT CAG G-3′) and DLK1FS (5′-AGG CAC CTG CGT GGA TGA T-3′). For quantitative real-time PCR, total RNA from cell lines was extracted with the RNAzol B (Biogenesis, Poole, UK) according to the manufacturer's instruction and treated with DNAse 1 (Invitrogen, Paisley, UK). Total RNA (1 *μ*g) was reverse transcribed as described previously. The cDNA was used in triplicate in a real-time PCR analysis using a real-time Thermal Cycler Model 7900 (Applied Biosystems). The primer sets are: *DLK1* (5′-GCG AGG ATG ACA ATG TTT GC-3′ (forward) and 5′-AGC AGG CCC GAA CAT CTC TA-3′ (reverse)) and *GTL2* (5′-ATC AGC CAA GCT TCT TGG AA-3′ (forward) and 5′-AGC TTC CAT CCG CAG TTC T-3′ (reverse)). Beta-actin was used as a control for normalisation. PCR was performed in a 25 *μ*l volume that included 12.5 *μ*l of 2 × Cyber green (Applied Biosystems), 25 ng of cDNA template and 0.0125–0.025 pmol of each primer. Relative RNA quantification was performed using the comparative *C*_T_ method. The fold differences in RNA expression are the mean value from three independent observations.

### Treatment of cell lines with 5-aza-2′-deoxycytidine (5-aza-dC)

5-Aza-dC (Sigma, Gillingham, Dorset, UK) was freshly prepared in ddH_2_O at 2 mg ml^−1^ and filter sterilised. A total of 1 × 10^6^ cells were plated in 75 cm^2^ flask in RPMI 1640 medium supplemented with 10% FCS and left to settle for 24 h (day 0). Cells were treated with 2 *μ*M of 5-aza-dC at day 1 and 4 and harvested at day 5. The culture medium was changed before each treatment and 24 h after treatment.

## RESULTS

### Epigenetic analysis of *DLK1/GTL2* domain in neuroblastoma tumours and cell lines

The methylation status of the *GTL2* promoter DMR was assessed in four normal control blood DNA samples and all four cases demonstrated both methylated and unmethylated allele products. We then analysed tumour DNA from 20 primary neuroblastomas. We found that five of 20 (25%) neuroblastoma tumours demonstrated only products from a methylated allele at the *GTL2* promoter DMR. The other 15 tumours showed a normal result with methylated and unmethylated allele products. In four of five tumours with hypermethylation of the *GTL2* promoter DMR, heterozygosity at a *DLK1* SNP (c.564T>C) (*n*=−2) or at closely linked microsatellite markers (D14S598, D14S1426, D14S749 and D14S1006) excluded 14q32 maternal allele loss as a cause of the *GTL2* promoter DMR hypermethylation.

**Figure 1:**
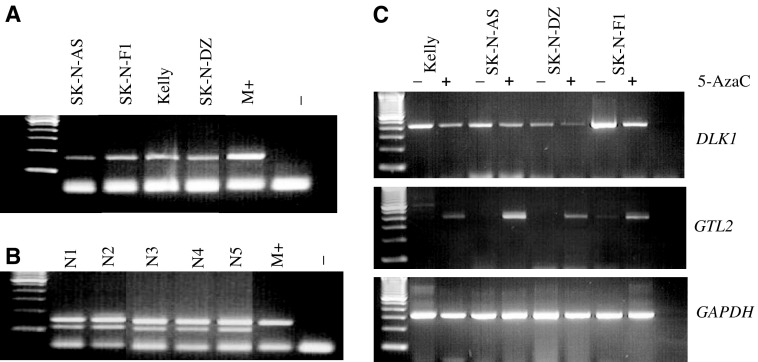
(**A**) Methylation-specific multiplexed PCR of the DMR upstream *GTL2* promoter in neuroblastoma cell lines showing the presence of only methylated allele of 160 bp. M+ is *in vitro* methylated DNA. (**B**) Normal blood DNA was used as control showing both unmethylated (120 bp) and methylated (160 bp) allele. (**C**) RT–PCR analysis of *DLK1* and *GTL2* in four neuroblastoma cell lines before (−) and after (+) treatment with demethylating agent 5-AzaC. *DLK1* expression was reduced, while *GTL2* expression was activated following 5-AzaC treatment. Molecular weight marker was a 100-bp ladder (Invitrogen).

**Figure 2:**
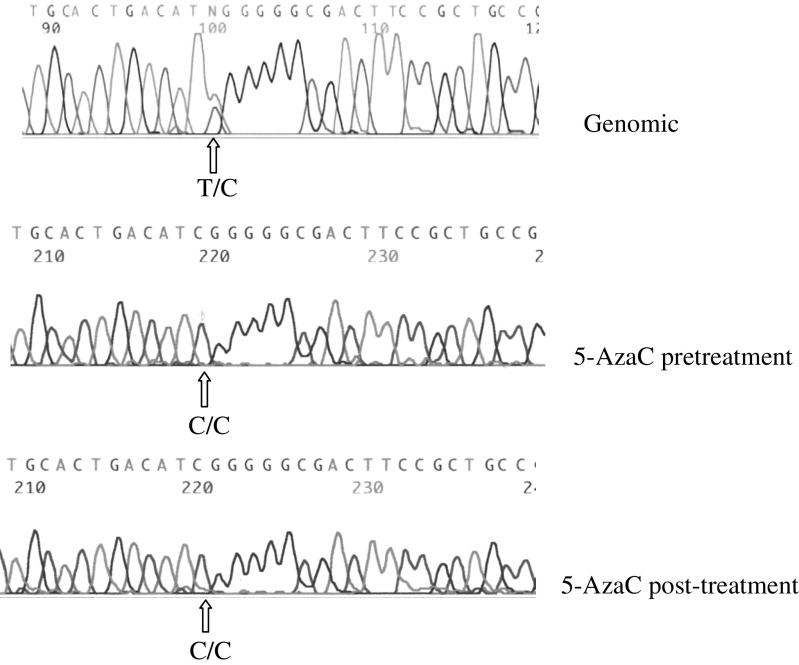
Analysis of *DLK1* allele expression in the informative SK-N-AS cell line. *DLK1* is monoallelically expressed before and following treatment with 5-AzaC.

In order to investigate whether *GTL2* promoter DMR hypermethylation in tumours was associated with alterations in *DLK* and *GTL2* expression and imprinting, we examined the *GTL2* promoter DMR methylation status in four neuroblastoma cell lines (SK-N-F1, SK-N-AS, SK-N-DZ, Kelly). All four neuroblastoma cell lines demonstrated *GTL2* promoter DMR hypermethylation (Figure 1A and B). One cell line, SK-N-AS, was heterozygous for a transcribed *DLK1* SNP, thus enabling us to (a) exclude maternal allele loss as the cause of 5′ *GTL2* DMR hypermethylation and (b) perform allele-specific expression studies to investigate *DLK1* imprinting status. We also analysed *DLK1* and *GTL2* expression before and after treatment with the demethylating agent 5-azacytidine (5-AzaC) in the four neuroblastoma cell lines with *GTL2* promoter DMR hypermethylation. In all cases, there was silencing of *GTL2* expression in the untreated cell line and reactivation of *GTL2* expression after treatment with 5-AzaC (Figure 1C and Table 1). In addition, 5-AzaC treatment resulted in a reduction in *DLK1* mRNA expression. Analysis of *DLK1* allelic expression in the informative SK-N-AS neuroblastoma line demonstrated monoallelic (allele C) *DLK1* expression pre- and post-treatment with 5-AzaC (Figure 2). Thus, hypermethylation of the *GTL2* promoter DMR in the SK-N-AS was associated with the upregulation of *DLK1* expression and silencing of *GTL2* expression, but not LOI of *DLK1*.

**Figure 3:**
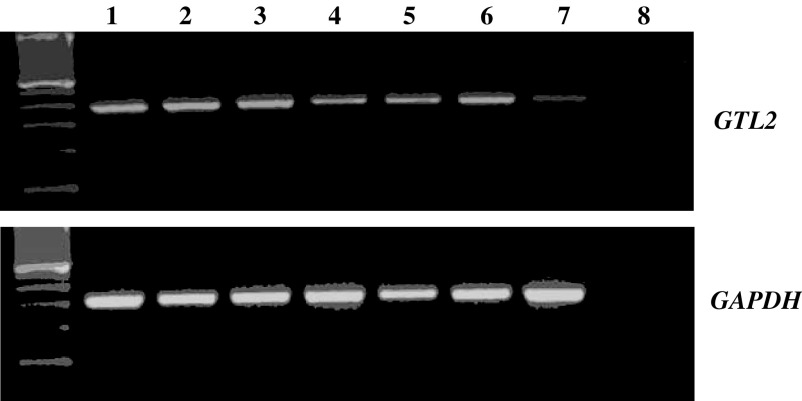
RT–PCR showing *GTL2* expression in neuroblastoma tumours without gain of methylation at 5′ *GTL2* DMRs (lanes 1–6) and tumour with gain of methylation at 5′ *GTL2* DMRs (lane 7). Low level of *GTL2* expression in this tumour may be caused by normal tissue contamination. Lane 8 is a negative control and *GAPDH* was used as unlinked control gene.

Hypermethylation of 5′ *GTL2* promoter was observed in five of 20 (25%) of neuroblastoma tumours. RNA was available from seven of the 20 tumours (see Figure 3). A low level of *GTL2* expression was detected in the tumour with hypermethylation of the 5′ *GTL2* promoter DMR (possibly caused by normal tissue contamination).

**Figure 4:**
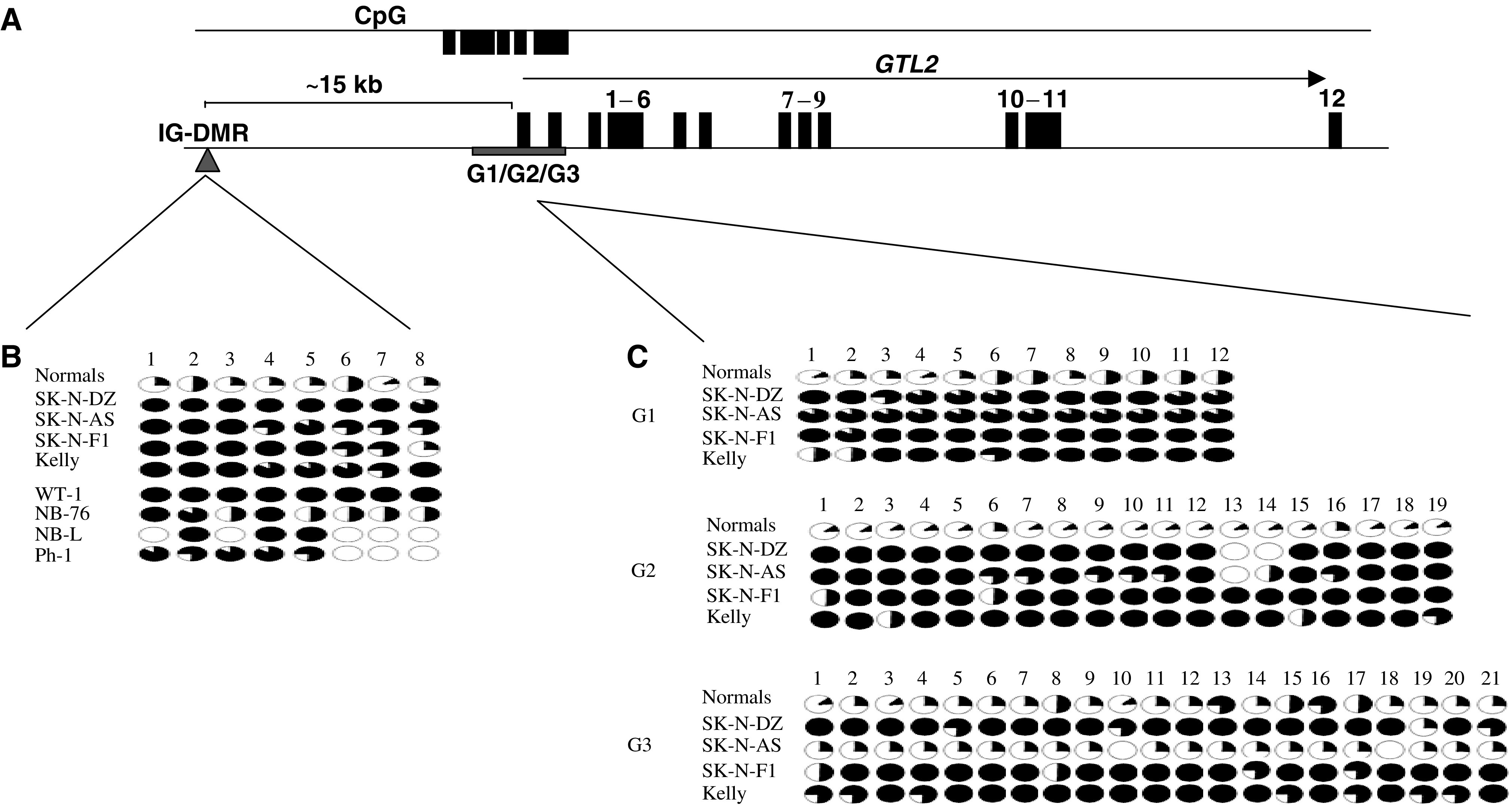
(**A**) Schematic representation of *GTL2* and IG-DMRs in human showing the position of regions analysed: G1=−363 bp → −161 bp upstream, G2=+293 bp → +673 bp downstream, and G3=+1213 bp → +1583 bp downstream of *GTL2* transcription start site, GeneBank accession no. AY314975; IG-DMR=nt 51021–51180, GeneBank accession no. AL 117190). (**B**) Methylation status of the CpGs in the IG-DMR in the normal blood, neuroblastoma cell lines, a Wilms' tumour, two neuroblastoma tumours and a phaeochromocytoma tumour with gain of methylation at the 5′ *GTL2* DMRs. (**C**) Methylation status of *GTL2* DMRs in neuroblastoma cell lines. The methylation status is shown in circles. Open and filled circles indicates complete unmethylation and full methylation, respectively. Partially filled circles indicate the degree of partial methylation.

To establish the precise epigenetic status of individual CpGs within the *GTL2* promoter DMR region, bisulphite sequencing was undertaken in four normal control blood DNA samples and in the SK-N-AS hypermethylated neuroblastoma cell line, which is heterozygous for *DLK1* SNP. A total of 54 CpGs were analysed in three subregions of the CpG-rich region upstream of *GTL2* (G1 *n*=12 CpGs, G2 *n*=19 and G3 *n*=21). Between eight and 10 clones were sequenced from each of the normal blood samples and the methylation status of each of the 54 CpGs determined (see Figure 4C). Comparison of the CpG methylation status of the *GTL2* promoter DMR subregions in controls and the neuroblastoma cell line revealed that within subregions G1 and G2, there was extensive methylation of CpGs in SK-N-AS compared to normal control DNAs. However, in subregion G3, there was little difference between the CpG methylation status of normal control DNAs and the SK-N-AS cell line DNA.

### Methylation analysis of the *GTL2* promoter DMR in phaeochromocytoma and Wilms' tumour

Methylation analysis of the *GTL2* DMR was then undertaken in 20 phaeochromocytomas and 40 Wilms' tumours. *GTL2* promoter DMR hypermethylation was detected in two phaeochromocytomas (10%) and one Wilms' tumour (2.5%). Genotyping with 14q32 microsatellite markers (D14S598, D14S1426, D14S1006, D14S749) excluded 14q32 allele loss in all three tumours.

### Methylation analysis of IG-DMR

Recently, an IG-DMR was demonstrated to function as an imprinting control element for all imprinted genes on the maternal chromosome only (Lin *et al*, 2003). To determine whether *GTL2* promoter DMR hypermethylation was associated with epigenetic alterations at the upstream IG-DMR, we analysed the methylation status of the eight CpGs within the IG-DMR in four normal control DNAs and in four neuroblastoma cell lines with 5′ *GTL2* DMR methylation. Eight to 12 individual clones for each were analysed for IG-DMR CpGs methylation status. In the four normal control samples, 32% of IG-DMR CpGs sequenced were methylated, while in the SK-N-AS neuroblastoma cell line with *GTL2* promoter DMR hypermethylation, all eight CpGs (*n*=12 clones) were methylated. In addition, the three neuroblastoma cell lines with 5′ *GTL2* DMR hypermethylation (but uninformative for *DLK1* imprinting status) showed almost complete methylation at all IG-DMR CpGs analysed (see Figure 4B). Similarly, analysis of the IG-DMR CpG methylation status in four primary tumours (two neuroblastoma, one phaeochromocytoma and one Wilms' tumour) with *GTL2* promoter DMR hypermethylation demonstrated heavy CpG methylation (although less complete than in the neuroblastoma cell lines, possibly because of normal tissue contamination). In contrast, cell lines and tumours with *GTL2* promoter DMR and IG-DMR hypermethylation did not show any differences from normal controls at the CpG-rich region upstream of the *DLK1* promoter nor at the two CpG-rich regions downstream of the *DLK1* promoter.

## DISCUSSION

We detected epigenetic changes within the *GTL2* promoter DMR (and the IG-DMR) in 25% of neuroblastoma tumours, 10% of phaeochromocytoma and 2.5% of Wilms' tumours analysed. In most cases, allele loss was excluded as a cause of the apparent hypermethylation. All cases with *GTL2* promoter DMR methylation also demonstrated hypermethylation at the upstream IG-DMR region, but epigenetic alterations were not detected in the three CpG-rich regions close to *DLK1*. Analysis of *DLK1* and *GTL2* expression in four neuroblastoma cell lines with *GTL2* DMR hypermethylation demonstrated repression of *GTL2* transcription and re-expression of *GTL2* after treatment with 5-AzaC. In most cell lines, re-expression of *GTL2* after treatment with 5-AzaC was associated with downregulation of *DLK1* expression. Furthermore, in an informative neuroblastoma cell line (SK-N-AS), there was no evidence of LOI of *DLK1*. The absence of LOI of DLK1 is consistent with other reports of monoallelic *DLK1* expression in neuroblastoma, brain tumours and lymphoma (van Limpt *et al*, 2003; Yin *et al*, 2004). The *DLK1/GTL2* imprinted domain has similarities to the *IGF2/H19* gene at 11p15.5. In both cases, two closely linked genes are reciprocally imprinted, and both *H19* and *GTL2* do not appear to encode a protein. Like *H19* and *Igf2*, *Gtl2* and *Dlk* transcripts can be found in the same tissues during development (Takada *et al*, 2000). In Beckwith–Wiedemann syndrome and sporadic Wilms' tumour, hypermethylation of the *H19* promoter DMR (and the upstream ‘CTCF box’ DMR) is associated with silencing of *H19* expression (Cui *et al*, 2001; Nakagawa *et al*, 2001). We identified hypermethylation of the *GTL2* DMRs in tumours and cell lines, so there are apparent similarities between epigenetics at the *H19* and *GTL2* loci in human neoplasia, although the frequency of *GTL2* hypermethylation in Wilms' tumour is much less than that at *H19*. However, whereas *de novo* hypermethylation of the *H19* promoter and CTCF box DMRs is associated with *H19* silencing and LOI of IGF2, LOI of *DLK1* was not associated with *GTL2* promoter DMR and IG-DMR hypermethylation. In mice, deletion of the unmethylated copy of the IG-DMR leads to bidirectional LOI of all genes in the imprinted cluster after maternal inheritance, but imprinting is unaltered after paternal transmission of the deleted methylated copy (Lin *et al*, 2003). This asymmetric regulation of imprinting distinguishes the imprinting control mechanisms in the 11p15.5 and the 14q32 imprinted gene clusters. The Callipyge phenotype demonstrates complex inheritance patterns. Thus, while paternal inheritance of the Callipyge mutation is associated with muscle hypertrophy, and maternal transmission is not, surprisingly, homozygotes do not demonstrate the phenotype. This unusual inheritance pattern (known as polar overdominance) has been attributed to the Callipyge mutation affecting a long-range control element resulting in enhanced expression *in cis* of all genes in the heterozygote sheep (but with no change in their imprinting) (Charlier *et al*, 2001; Freking *et al*, 2002; Georges *et al*, 2003). Furthermore, the absence of a mutant phenotype in the homozygotes suggests that overexpression of imprinted genes on the maternal chromosome abrogates the functional defect caused by the paternal chromosome *in trans*. Hence, when the Callipyge mutation is paternally inherited, *DLK1* transcription is upregulated, while *GTL2* is upregulated after maternal transmission (Charlier *et al*, 2001; Freking *et al*, 2002; Georges *et al*, 2003). In homozygotes, it is postulated that *GTL2*-associated transcripts negatively regulate *DLK1* transcripts; however, the precise mechanism is not known. Our finding that hypermethylation of the *GTL2* promoter and upstream DMR is associated with silencing of *GTL2* and upregulation of *DLK1* without LOI would be consistent with a model, whereby *GTL2*-associated transcripts negatively regulate *DLK1* transcription and further analysis of neuroblastoma cell lines with GTL2 hypermethylation and silencing may provide important insights into the mechanisms of imprinting control in the 14q32 imprinted domain.

*DLK1* encodes a transmembrane protein with six EGF-like repeats that shows homology to the *Delta* gene in *Drosophila melanogaster*, which is involved in the Notch signalling pathway. The precise function of the DLK1 protein is unclear, but *DLK1* expression is upregulated in myelodysplastic syndrome, a slowly progressing haematological malignancy, and in uterine leiomyomata (compared to normal myometrium) (Miyazato *et al*, 2001; Tsibris *et al*, 2002). In addition, overexpression of the *DLK1* protein is reported to prevent adipocyte differentiation of 3T3-L1 cells in response to IGF1 or insulin. These latter effects were attributed to changes in the activation levels and kinetics of extracellular-regulated kinase/mitogen-activated protein kinase (Ruiz-Hidalgo *et al*, 2002). However, although it might be postulated that increased DLK1 expression might be pro-oncogenic, there is no direct evidence for this. Indeed, van Limpt *et al* (2003) suggested that neuroblastomas DLK1 expression increases during chromaffin cell lineage differentiation and high DLK1 expression in neuroblastoma cell lines may merely indicate that the neuroblastoma has developed from a later stage chromaffin cell precursor. Hence, upregulation of DLK1 expression associated with *GTL2* promoter and intergenic DMR hypermethylation may be coincidental to tumorigenesis. *GTL2* promoter and intergenic DMR hypermethylation was associated with transcriptional silencing of *GTL2* and treatment with 5-AzaC resulted in re-expression of *GTL2*. Recently, an isoform of *GTL2*, *MEG3a*, was reported to be expressed highly in normal pituitary and brain and in other tissues including adrenal (Zhang *et al*, 2003), but not in many pituitary tumours. Furthermore, transfection of MEG3a into a HeLa cell line inhibited cell proliferation in the *in vitro* colony formation and growth rate assays. Hence, although neither GTL2 nor MEG3a have been shown to encode an expressed protein, *GTL2* promoter and intergenic DMR hypermethylation might promote tumorigenesis by downregulating *GTL2* expression. These findings are similar to those reported for *H19* (Hao *et al*, 1993), although the precise role of H19 silencing *per se* in Wilms' tumour and tumour susceptibility in Beckwith–Wiedemann syndrome has not been unequivocally established (Tycko, 2000). Thus, further investigations of the potential roles of *GTL2*, *MEG3a* and epigenetic changes in the *DLK1*/*GTL2* domain are indicated.

## Figures and Tables

**Table 1:** *DLK1* and *GTL2* expression in neuroblastoma cell lines following 5-AzaC treatment analysed by real-time PCR

**Cell line**	***DLK1* Fold change (±s.d.)^a^ after 5-AzaC treatment**	***GTL2* Fold change (±s.d.)^a^ after 5-AzaC treatment**
SK-N-DZ	0.282±0.056	5.437±2.037
SK-N-AS	0.219±0.097	3.953±0.724
SK-N-F1	0.365±0.259	2.773±0.570
Kelly	0.173±0.015	2.693±0.825

5-AzaC=5-azacytidine; PCR=polymerase chain reaction; s.d.=standard deviation.

a The fold differences in RNA expression are the mean±s.d. of three independent observations.
